# Technical and tactical diagnosis model of table tennis matches based on BP neural network

**DOI:** 10.1186/s13102-021-00283-3

**Published:** 2021-05-20

**Authors:** Wenwen Huang, Miaomiao Lu, Yuxuan Zeng, Mengyue Hu, Yi Xiao

**Affiliations:** 1 China Table Tennis College, Shanghai University of Sport, Shanghai, 200438 China; 2 School of Economics and Management, Shanghai University of Sport, Shanghai, China

**Keywords:** Artificial neural network, Table tennis, Techniques and tactics, Diagnostic model, Winning probability

## Abstract

**Background:**

The technical and tactical diagnosis of table tennis is extremely important in the preparation for competition which is complicated by an apparent nonlinear relationship between athletes’ performance and their sports quality. The neural network model provides a high nonlinear dynamic processing ability and fitting accuracy that may assist in the diagnosis of table tennis players’ technical and tactical skill. The main purpose of this study was to establish a technical and tactical diagnosis model of table tennis matches based on a neural network to analyze the influence of athletes’ techniques and tactics on the competition results.

**Methods:**

A three-layer Back Propagation (BP) neural network model for table tennis match diagnosis were established. A Double Three-Phase evaluation method produced 30 indices that were closely related to winning table tennis matches. A data sample of 100 table tennis matches was used to establish the diagnostic model (*n* = 70) and evaluate the predictive ability of the model (*n* = 30).

**Results:**

The technical and tactical diagnosis model of table tennis matches based on BP neural network had a high-level of prediction accuracy (up to 99.997%) and highly efficient in fitting (*R*^*2*^ = 0.99). Specifically, the technical and tactical diagnosis results indicated that the scoring rate of the fourth stroke of Harimoto had the greatest influence on the winning probability.

**Conclusion:**

The technical and tactical diagnosis model of table tennis matches based on BP neural network was highly accurate and efficiently fit. It appears that the use of the model can calculate athletes’ technical and tactical indices and their influence on the probability of winning table tennis matches. This, in turn, can provide a valuable tool for formulating player’s targeted training plans.

**Supplementary Information:**

The online version contains supplementary material available at 10.1186/s13102-021-00283-3.

## Background

Compared with other physical events (such as track and field, cycling, swimming, etc.), one of the most important characteristics of ball games is that there is a nonlinear relationship between athletes’ performance and their sports quality [1]. When sports are classified into five levels according to their technical and tactical importance, and ball games are highest on the list as a successful outcome is predicated on the combined use of techniques and tactics [2]. Therefore, being able to diagnose athletes’ technical and tactical performance is extremely important not only for training but preparation for competition.

Table tennis is very popular and suitable for most people. The current table tennis match is an 11-point system, with the two sides serving alternately and two serves per round. Compared with other ball games, table tennis has the characteristics of many variations, fast ball speed, and fast spin speed, which requires a high skill level for professional table tennis players. The outcomes of table tennis competitions are affected by multiple factors, such as physical fitness, psychology, techniques, and tactics. Perhaps the most influential of these are techniques and tactics which appear to have a direct effect on the competition results [3]. The technical and tactical diagnosis of table tennis competitions can not only provide useful guidance for training but also improve the competitive ability of athletes [4, 5]. Therefore, the systematic analysis and diagnosis of the players’ technical and tactical characteristics are very important and crucial in preparing athletes for table tennis competitions [6, 7].

With regard to the technical and tactical diagnosis methods of table tennis, the most well-known is Wu and colleagues’ three-phase evaluation method [8]. This approach uses a player’s last stroke in each rally as the observation unit and divides each rally into three phases: (a) attack after service (the first and third strokes), (b) attack after receive (the second and fourth strokes), and (c) stalemate phase (after the fourth strokes). The scoring rate (SR) and the usage rate (UR) are used as evaluation indices for analysis. This method has been widely used in many studies to evaluate the strength of table tennis players’ techniques and tactics. Practically, the top table tennis teams such as the People’s Republic of China’s national team uses this method to conduct technical and tactical analyses for their own players and major opponents while preparing for the World Championships and Olympic Games [7, 9–11].

A table tennis game can be divided into three stages according to the time sequence characteristics of a game: (a) beginning, (b) middle, and (c) end. In 2018, Xiao and his colleagues posited that a more detailed analysis may result by combining the three-phase evaluation method and the three stages of a game [12]. Calling it the Double Three-Phase, this approach still uses the SR and UR indices but calculates them for the beginning, middle, and end stages of each game. Thus, coaches and athletes are provided a more fine-grain analysis to evaluate the strength of a player’s technical and tactical performance in terms of variation of use and at what points in the match [12]. This provides a more comprehensive analysis of the variation and strength of players’ techniques and tactics in the competition, which is a useful supplement to the three-phase evaluation method.

With the development of information technology, there are a variety of diagnosis and analysis methods for game performance, such as the Multiple Liner Regression analysis, the Markov Chain, and the Artificial Neural Network (ANN) [7]. Given that table tennis matches are affected by many factors, and the relationship between the competition result and players’ technical and tactical ability is mainly nonlinear [13], the use of these linear analyses methods makes it difficult to determine the impact of athletes’ technical and tactical performance on the result. Therefore, the two-fold purpose of this study was to: (a) develop a computational model based on a nonlinear model that can simulate the winning probability of a table tennis match, and (b) apply the model to analyze the technical and tactical diagnosis of a world’s elite table tennis player based on the Double Three-Phase evaluation method. Through changing of index weights in the diagnostic model, the players’ strengths and weaknesses in techniques and tactics could be found, which provided a valuable reference for coaches to formulate targeted training plans and to adjust the technical and tactical strategy in matches, and for players, it is helpful to overcome the shortcomings in techniques and tactics and improve their table tennis technique levels.

### Literature review

A Multiple Linear Regression analysis model can be used to diagnose the relationship between sports competition performance and a series of influencing factors [14]. One advantage of a multiple line regression model is that it can better reveal the impact of each independent variable on the dependent variable. However, the multiple line regression analysis model requires close linear correlation between independent variables and dependent variables, therefore, making it difficult to diagnose technical and tactical use in table tennis matches [15].

The Markov Chain model can simulate the winning probability of a table tennis match. When one of the indices affecting the win of a match changes, the model can diagnose the winning probability of a match again. Hence, researchers can determine what influence a change in one index value (increasing or decreasing) will have on the output (winning probability) by comparing the difference of winning probability. Pfeiffer et al. proposed four different state transition models to describe the tactical behavior of table tennis by using the Markov Chain, and the results demonstrated that the random path simulation of the Markov Chain was valuable in the technical and tactical diagnosis of table tennis matches [16]. Newton used the stochastic Markov Chain model to propose the probability density function of players’ winning in tennis matches, and the results showed that the Markov Chain could provide more detailed information for the diagnosis of players’ athletic ability and the winning probability of tennis matches [7, 17].

An Artificial Neural Network (ANN) is a complex network system that carries out parallel processing and nonlinear transformation of information by simulating human brain neural processing. Compared with traditional multiple linear regression, ANN has a high nonlinear dynamic processing ability [18]. The basic idea of a neural network model is quite similar to that of the Markov Chain model but the latter requires the integrity of data structure and its time sequence while the ANN has no strict requirement for input indices [7]. When the input and output relationship is too complicated to be expressed by general formulas, it is easy for neural networks to realize their highly nonlinear mapping relationship. The learning process of a neural network is essentially a process of optimizing the weights of the neural network, which often requires hundreds of iterative operations. Hence, choosing a reasonable neural network structure can reduce network training times and improve the training accuracy of the model. ANNs have been widely used in the fields of pattern recognition, automatic control, and economic diagnosis, etc. [19–22].

A possible option to these three analysis methods is a Back Propagation (BP) neural network. A BP neural network is a multi-layered feed forward neural network trained by backward error propagation [23]. This algorithm proposed by Rumelhart and McClelland uses squared error as its objective function and calculates the minimum of the error function by gradient descent method [7]. The basic structure of a three-layer BP neural network includes three layers: (a) input, (b) hidden, and (c) output. The key to establishing a three-layer BP network model is the determination of the input layer’s neuron number, the hidden layer’s number, the hidden layer’s neuron number, the selection of transferring function as well as the training function. The high nonlinear processing ability of BP neural network makes it widely applied in game prediction, and techniques and tactics diagnosis for ball matches [13, 24, 25]. Yang & Zhang used BP neural network and multiple regression to analyze the technical and tactical ability of elite male table tennis players, and the results showed that the overall fitting accuracy of the BP neural network was higher than that of the multiple regression model [14].

At present, some potential innovative approaches have been proposed and applied in the outcome prediction of other sports, e.g. applying passing network indicators to predict the winning probability of football games, or introducing the negative Poisson binomial distribution to assess the probabilities of all possible outcomes of a darts tournament [26, 27].

Given that the BP neural network model has no strict requirement for input indices and has high fitting accuracy, the use of the BP neural network seems appropriate when attempting to establish a technical and tactical diagnosis model of table tennis matches. When one of the technical and tactical index’s value is adjusted, the winning probability of the match can be recalculated through the diagnostic model in order to examine the impact of that index on the winning probability. Thus, this model was considered to be applied in this study.

## Method

### Three-phase evaluation method

The Three-Phase evaluation method of table tennis technique and tactic divides each rally into three phases: the serve and attack phase, the receive and attack phase, and the stalemate phase. It uses the scoring rate (*SR*) and the usage rate (*UR*) in each phase of each game to analyze the strength of table tennis players’ techniques [7]. The definition of *SR* and *UR* were as follows [4]:

Usage Rate: $$ {UR}_{\mathrm{i}}=\frac{{\mathrm{W}}_{\mathrm{i}}+{\mathrm{L}}_{\mathrm{i}}}{S} $$

Scoring Rate: $$ {SR}_{\mathrm{i}}=\frac{{\mathrm{W}}_{\mathrm{i}}+{\mathrm{L}}_{\mathrm{i}}}{S} $$

where *i* is the number of each phase, *UR*_*i*_ is the usage rate at the *i*-th phase, *SR*_*i*_ is the scoring rate at the *i*-th phase, *W*_*i*_ is the scoring points at the *i*-th phase, and *L*_*i*_ is the losing points at the *i*-th phase, and S is the total points of both sides scored during a match.

### Division of a game

According to the time sequence characteristics of each game, a table tennis game could be divided into three stages: the beginning, the middle, and the end, which were defined as follows: (a) the beginning of a game: before the score of either side reaches 5 points (first to fourth points), (b) the middle of a game: from the score of either side reaches 5 points to 8 points (fifth to eighth points), and (c) the end of a game: from the score of either side reaches 9 points to the end of a game.

### Double three-phase evaluation method

Combining the Three-Phase evaluation method with the division of a game, Xiao et al. put forward the Double Three-Phase evaluation method, which still used the SR and UR in the beginning, middle, and the end of a game to evaluate the strength of players’ technical and tactical performance [12].

### The analysis indices based on the double three-phase evaluation method

In this study, the 30 technical and tactical analysis indices which have a closer relation to winning a competition were selected based on the Double Three-Phase evaluation method, including three kinds of scoring rate and usage rate, which were illustrated in Table 1.

**Table 1:** The 30 technical and tactical analysis indices based on the double three-phase evaluation method

	The beginning of a game		The middle of a game		The end of a game	
Phases	Bh	Name	Bh	Name	Bh	Name
serve and attack phase	X1	UR of Serve	X3	UR of Serve	X5	UR of Serve
	X2	SR of Serve	X4	SR of Serve	X6	SR of Serve
	X13	UR of the third stroke	X15	UR of the third stroke	X17	UR of the third stroke
	X14	SR of the third stroke	X16	SR of the third stroke	X18	SR of the third stroke
receive and attack phase	×7	UR of Receive	X9	UR of Receive	X11	UR of Receive
	X8	SR of Receive	X10	SR of Receive	X12	SR of Receive
	X19	UR of the fourth stroke	X21	UR of the fourth stroke	X23	UR of the fourth stroke
	X20	SR of the fourth stroke	X22	SR of the fourth stroke	X24	SR of the fourth stroke
stalemate phase	X25	UR after the fourth strokes	X27	UR after the fourth strokes	X29	UR after the fourth strokes
	X26	SR after the fourth strokes	X28	SR after the fourth strokes	X30	SR after the fourth strokes

### Definition of the competition result

In this paper, the player’s competition result of a match was described by the winning probability, which was defined as the total winning points of one side divided by the total scores of both sides in a match [6].

### The calculation of the weights of technical and tactical indices on winning probability

In this study, the impact of technical and tactical indices on the winning probability was examined by adjusting the value of each technical and tactical index, while keeping the other indices’ value unchanged, and recalculating the winning probability of the match through the diagnostic model [7].

The calculation steps of the weight of each technical and tactical index’s impact on the winning probability were as follows:

1. Inputting the unchanged data matrix into the neural network diagnostic model to training, and calculating the winning probability (wp1) of the matches.
2. According to the Formula (1), adjusting the value of the selected technical and tactical index, and keep the others unchanged [28].

1 $$ \mathrm{y}=0.238\ast \cos\ \left(\hbox{-} 1.32\ast \mathrm{x}+0.66\right)\hbox{-} 0.178 $$

where x is the unchanged value of the selected index, and y is the adjusted increment.

(3) Inputting the changed data matrix into the neural network diagnostic model to training again, and recalculating the winning probability (wp2) of the matches.
(4) Calculating the weight of selected technical and tactical index on the winning probability: weight = (wp1 ‐ wp2)/wp1 ∗ 100%. The larger the absolute weight value is, the higher the influence of the selected index on the winning probability.

#### Neural network diagnostic model

##### Neural network model structure

In this study, 100 matches of the world’s elite male table tennis players were selected as sample data. The selection criteria were: (a) ITTF world matches held from the year 2018 to 2020, (b) men’s singles, (c) elite table tennis player, and (d) the game video is complete and can play normally. The technical and tactical analysis of these matches was conducted by the research team using the Double Three-Phase evaluation method.

The descriptive statistics results of the 30 technical and tactical indices of these matches were shown in Table 2. The results of the technical and tactical analysis, including the 30 technical and tactical analysis indices and the winning probability, were used as the data sample, and a three-layer BP neural network model for table tennis match diagnosis was established by adopting the Levenberg-arquardt training function.

**Table 2:** Descriptive statistics of the 30 technical and tactical indices of the 100 matches

Variables	M ± SD	Variables	M ± SD	Variables	M ± SD
x1	.0645 ± .05043	x11	.1557 ± .0683	x21	.1973 ± .0607
x2	.7663 ± .4021	x12	.6379 ± .2378	x22	.4234 ± .2055
x3	.0620 ± .0458	x13	.2005 ± .0662	x23	.1975 ± .0611
x4	.8001 ± .3613	x14	.5617 ± .2431	x24	.3792 ± .1969
x5	.0567 ± .0400	x15	.2038 ± .0620	x25	.3917 ± .1060
x6	.8093 ± .3575	x16	.5376 ± .1879	x26	.4038 ± .1669
x7	.1577 ± .0661	x17	.2234 ± .0662	x27	.3861 ± .0957
x8	.6065 ± .2771	x18	.5527 ± .1813	x28	.3988 ± .1497
x9	.1507 ± .0583	x19	.1857 ± .0576	x29	.3667 ± .1079
x10	.6494 ± .2339	x20	.3912 ± .2355	x30	.3917 ± 1426

**Fig. 1:**
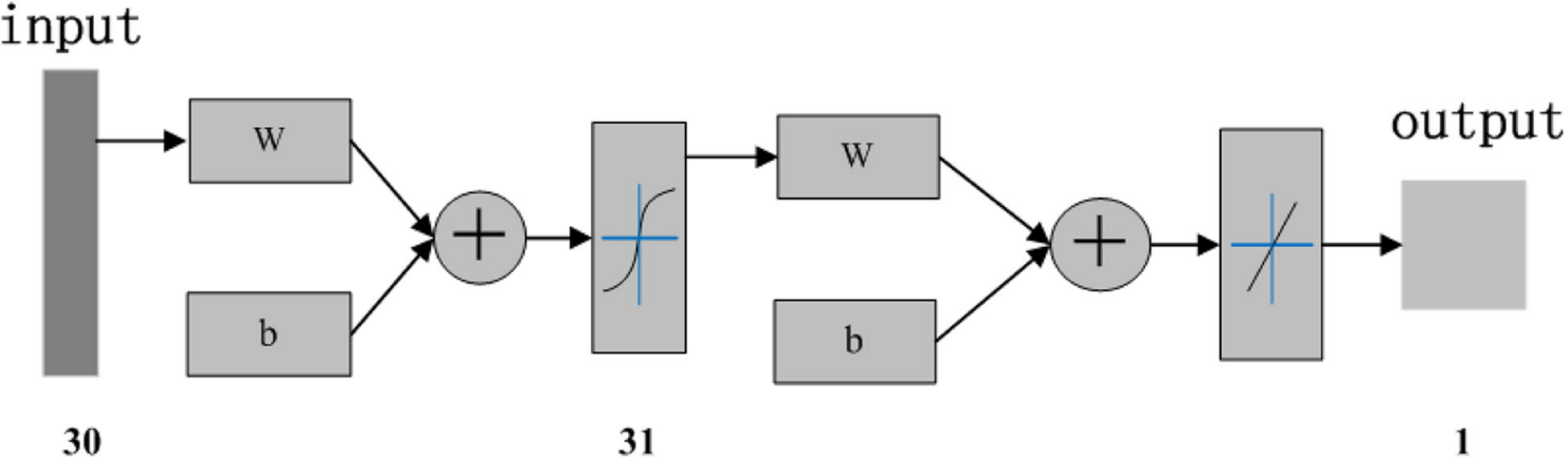
Neural network model structure

The input layer of the diagnostic model included 30 neurons which corresponded to 30 technical and tactical analysis indices (from × 1 to × 30), respectively. The output layer of the diagnostic model was the winning probability of a table tennis match, and the hidden layer included 31 neurons. The number of the hidden layer’s neurons can be changed according to the requirement of the model’s accuracy. The nonlinear S transferring function (activation function) tansig(n) was taken between the input layer’s neurons and the hidden layer’s neurons, and the linear transferring function purelin(n), i.e. f(x) = x, between the hidden layer’s neurons and the output layer’s neurons was taken, whose structure was shown in Fig. 1 [29].

##### Neural network model training

The 100 matches were randomly divided into two groups, of which 70 matches were taken as neural network training set to establish the diagnostic model of table tennis matches, and the other 30 matches were taken as the test set to evaluate the predictive ability of the diagnostic model.

The BP self-learning algorithm was used to learn the training samples. After learning, the winning probability (the output data) of the table tennis diagnostic model can be obtained. Then, the input and output data of the diagnostic model can reflect the functional relationship between the technical and tactical indicators (input layer) and the winning probability (output layer) of the matches. In order to make the diagnostic model have faster convergence speed and higher stability, the Levenberg-Marquardt training function was adopted in this paper. The Levenberg-Marquardt training rule had the characteristics of fast calculation speed and high precision, whose specific steps were as follow [29]:

- Step-1: Setting initial weight W;
- Step-2: For the input indices (× _1_, × _2_ ...... × _30_) of all training samples: (a) Calculating the output of hidden neurons: Hj = f (∑Wji Xi ‐ θj) (i = 1 to 30; j = 1 to 31), where W_ji_ is the connection weight between neuron i and j, θ_j_ is the *j*-th hidden neuron’s threshold value, (b) Calculating the output of the output neuron: y = f (∑Wj Hj ‐ θo) (j = 1 to 31), where W_j_ is the connection weight between neuron j and output neuron, θ_o_ is the output neuron’s threshold value, and (c) Calculating the mean squared error of all sample’s actual and expected output: e =  ∑ (t ‐ y)^2^/2 , where y is the actual output value, and t is the expected output value.
- Step-3: Anti-propagation computing weight correction vector: Δw = [J^T^J + uI]^‐1^J^T^e; Correcting all net weights: wk + 1 = wk + Δw, where k is training steps, J is Jacobian matrix derived by the partial derivative of the error to weight, I is a unit matrix, e is network error vector, and u is the increasing coefficient (a scalar). When the error has not reached the expected value, u is reduced, otherwise, u is increased.
- Step-4: Repeating Step-2 and Step-3 until one of the following conditions is met: the error meets the requirements or the training step exceeds the given value.

##### Neural network model programming

The training process of a neural network is essentially a process of optimizing the weights of the neural network, which requires hundreds of iterative operations, so the amount of calculation in the training process of the neural network is quite large and needs to be realized by programming. The neural network development kit of Matlab R2016b was utilized to develop the diagnostic model’s program. The pseudocode frame for the network training procedure was shown as following:

Input:

Input data to present to the network /* inputs of the model */

Target data defining network output /* targets of the model */

Randomly divide up the samples

Setting:

Input the number of the hidden neurons

Choosing a training algorithm

Training:

Training the model until meeting the requirement of the error setting

Output

## Results

The neural network diagnostic model was evaluated via the leave-one-out cross validation method, which is commonly used when dealing with a small number of samples [30].

### Training performance

**Fig. 2:**
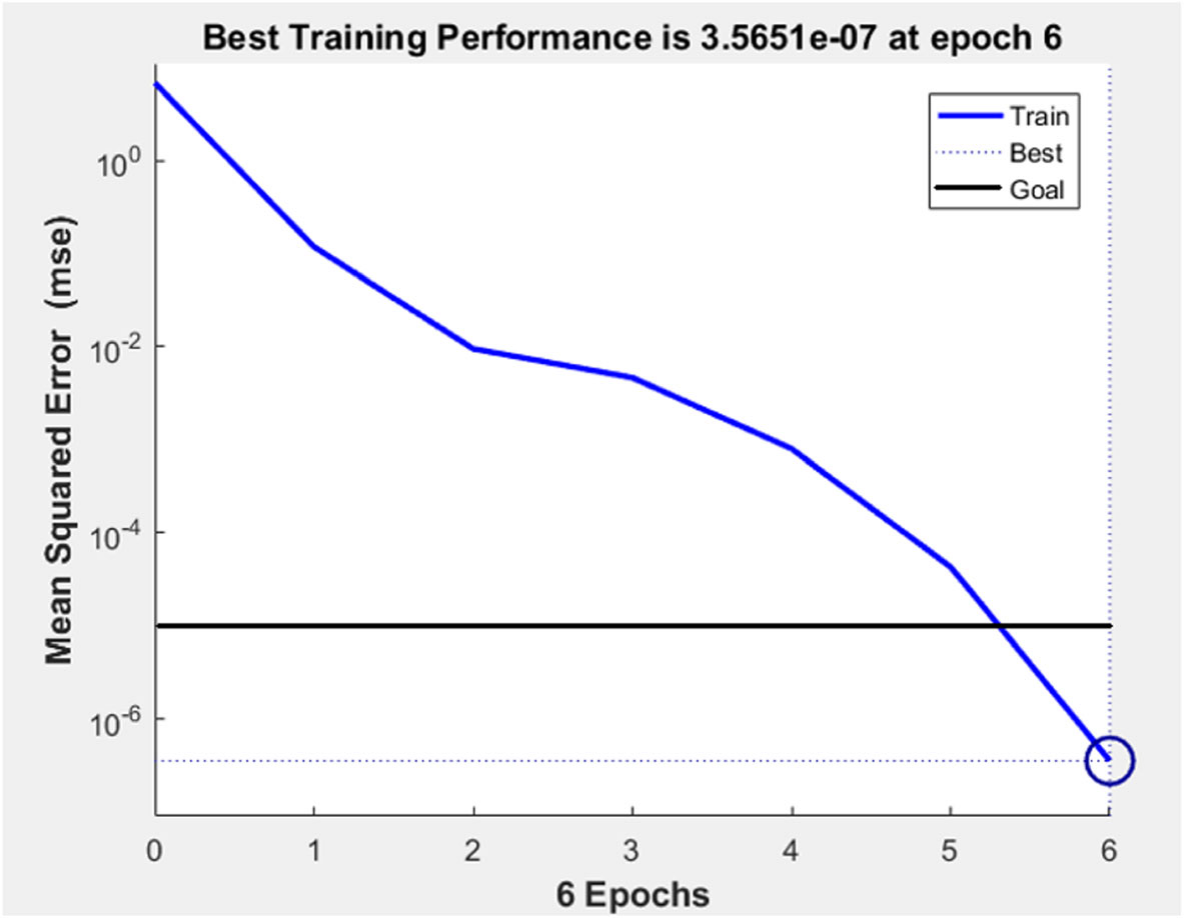
Training time and error

The training time (epochs) and mean squared error (MSE) of the table tennis diagnostic model were shown in Fig. 2. The x-axis is the training times (epochs) and the y-axis is the training error (MSE). The goal of the training error of the diagnostic model was set 1e-5. As shown in Fig. 2, when the training time reached 6 epochs, the model’s training error (3.57e-7) reached and exceeded the requirement (1e-5), which indicated the diagnostic model’s training performance was high.

#### Goodness-of-fit of the model

**Fig. 3:**
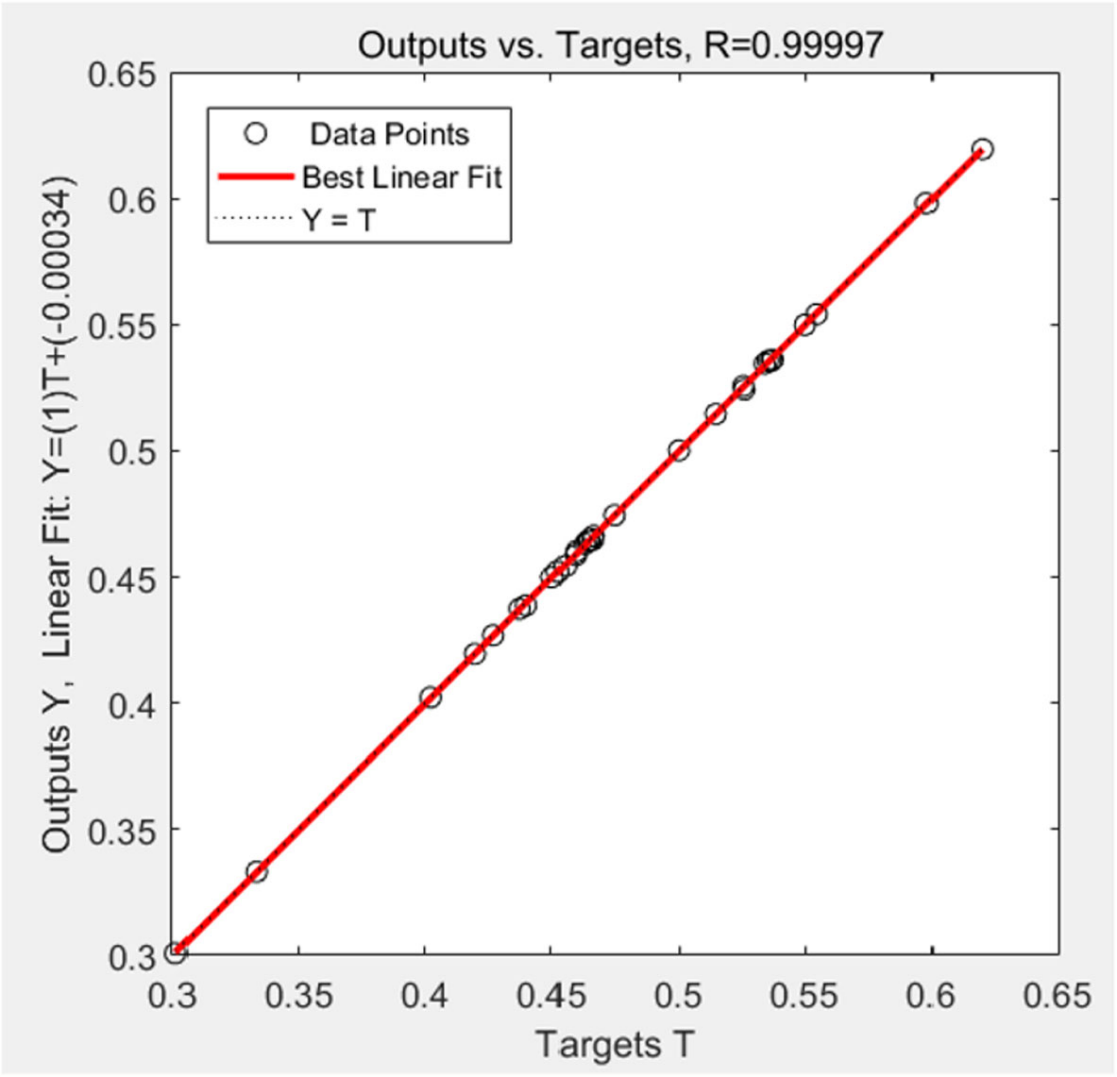
Goodness-of-fit of the model

After training and learning, the SIM function (program code: t_sim = sim (net, a)) was used to simulate the output of the model, and compare the output data with the observed values (targets) to evaluate the predictive ability of the diagnostic model. The result was shown in Fig. 3, in which the R value represented the correlation between outputs and targets (1 means a close relationship and 0 a random relationship), and the output data points (small white circle) of the neural network diagnostic model were all close to the dotted line, indicating that the network had a good performance and high goodness-of-fit (*R*^*2*^ = 0.99).

#### The prediction accuracy of the model

**Fig. 4:**
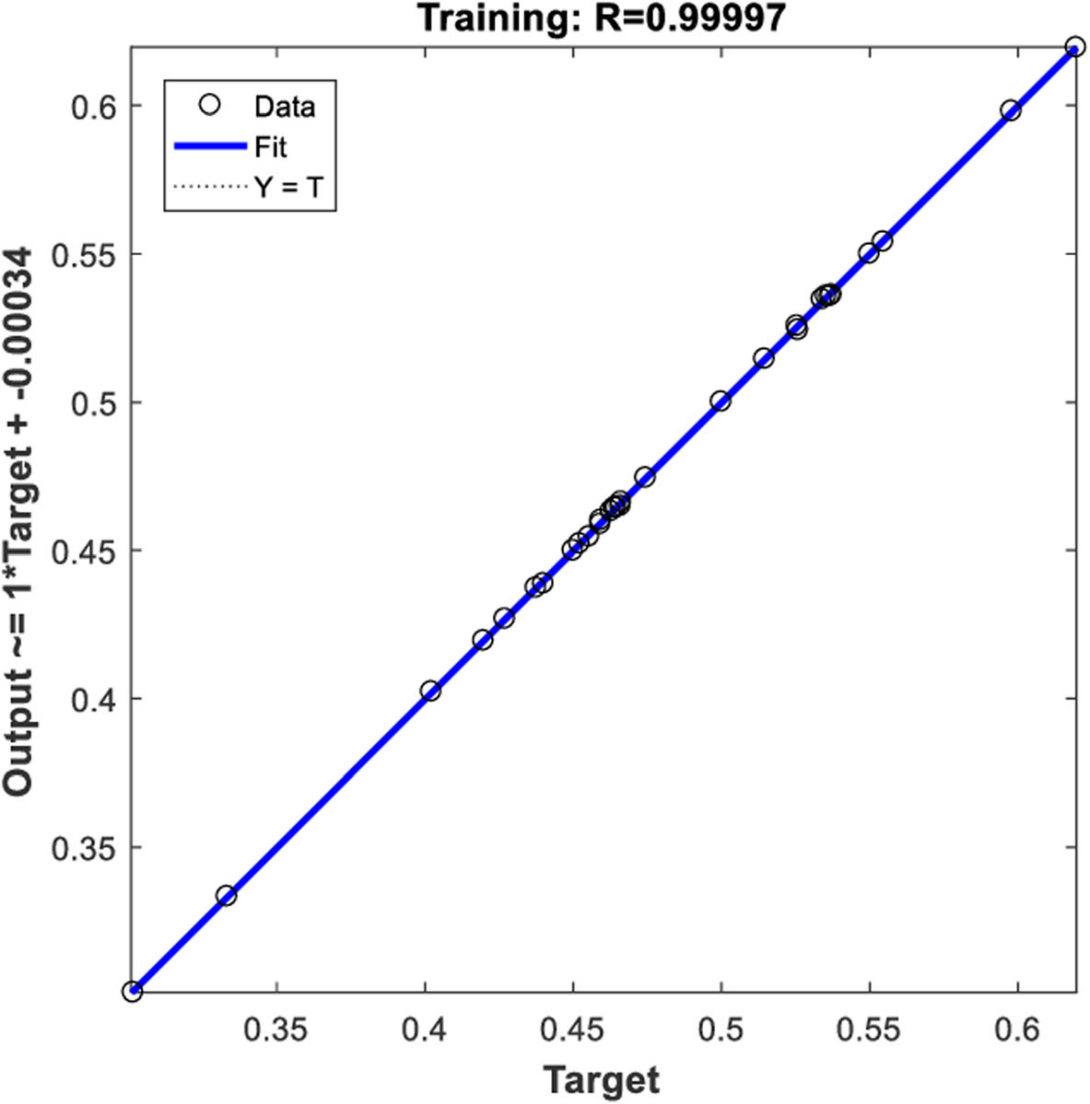
Accuracy of the diagnostic model

The 30 index data of the other 30 matches were input as test samples into the neural network diagnostic model of table tennis matches, and the simulated values of the winning probability of these matches were obtained. The maximum absolute error, the minimum absolute error, and the mean absolute error were calculated by comparing the simulated values of the model with the observed (real) values. These values were used to examine the model’s prediction accuracy, and the results were shown in Table 3 and Fig. 4.

**Table 3:** The error between actual and simulated values

Match No	Simulated value	Actual value	Error
No.1	0.452194	0.452200	0.000006
No.2	0.514612	0.514600	0.000012
No.3	0.333274	0.333300	0.000026
No.4	0.549943	0.550000	0.000057
No.5	0.474432	0.474500	0.000068
No.6	0.449924	0.450000	0.000076
No.7	0.426923	0.427000	0.000077
No.8	0.437415	0.437500	0.000085
No.9	0.466303	0.466200	0.000103
No.10	0.500112	0.500000	0.000112
No.11	0.402319	0.402200	0.000119
No.12	0.619540	0.619700	0.000160
No.13	0.525672	0.525500	0.000172
No.14	0.598103	0.597900	0.000203
No.15	0.464377	0.464600	0.000223
No.16	0.419550	0.419800	0.000250
No.17	0.463129	0.462800	0.000329
No.18	0.535736	0.535400	0.000336
No.19	0.464351	0.464000	0.000351
No.20	0.458779	0.459200	0.000421
No.21	0.554037	0.554600	0.000563
No.22	0.534597	0.534000	0.000597
No.23	0.300882	0.301600	0.000718
No.24	0.460113	0.459300	0.000813
No.25	0.454538	0.455400	0.000862
No.26	0.535627	0.536600	0.000973
No.27	0.536156	0.537200	0.001044
No.28	0.464899	0.466000	0.001101
No.29	0.438844	0.440000	0.001156
No.30	0.524326	0.525900	0.001574

The data of Table 3 showed that the maximum absolute error of the 30 matches was about 0.0016, while the minimum error was about 0.000006, the average error of the 30 matches was 0.0004. As shown in Fig. 4, the winning probability derived from the BP neural network diagnostic model of table tennis match was very close to the actual value, which showed that the diagnostic model based on BP neural network reached a high prediction accuracy up to 99.997% [14].

#### Application of the diagnostic model

In this paper, the Japanese famous table tennis player, Harimoto, was taken as an example. 20 international matches that Harimoto participated in in the past 5 years (from 2015 to 2019) and the technical and tactical analysis of the 20 matches was conducted using the double three-phase evaluation method. Then, the 30 technical and tactical analysis indices were input into the neural network diagnostic model for training and the outcome (winning probability) of the matches was obtained. The weights of 30 technical and tactical indices’ impact on the winning probability were also calculated by adjusting the value of each technical and tactical index one by one according to the Formula (1) and keep the others unchanged (repeat 30 times in total).

**Fig. 5:**
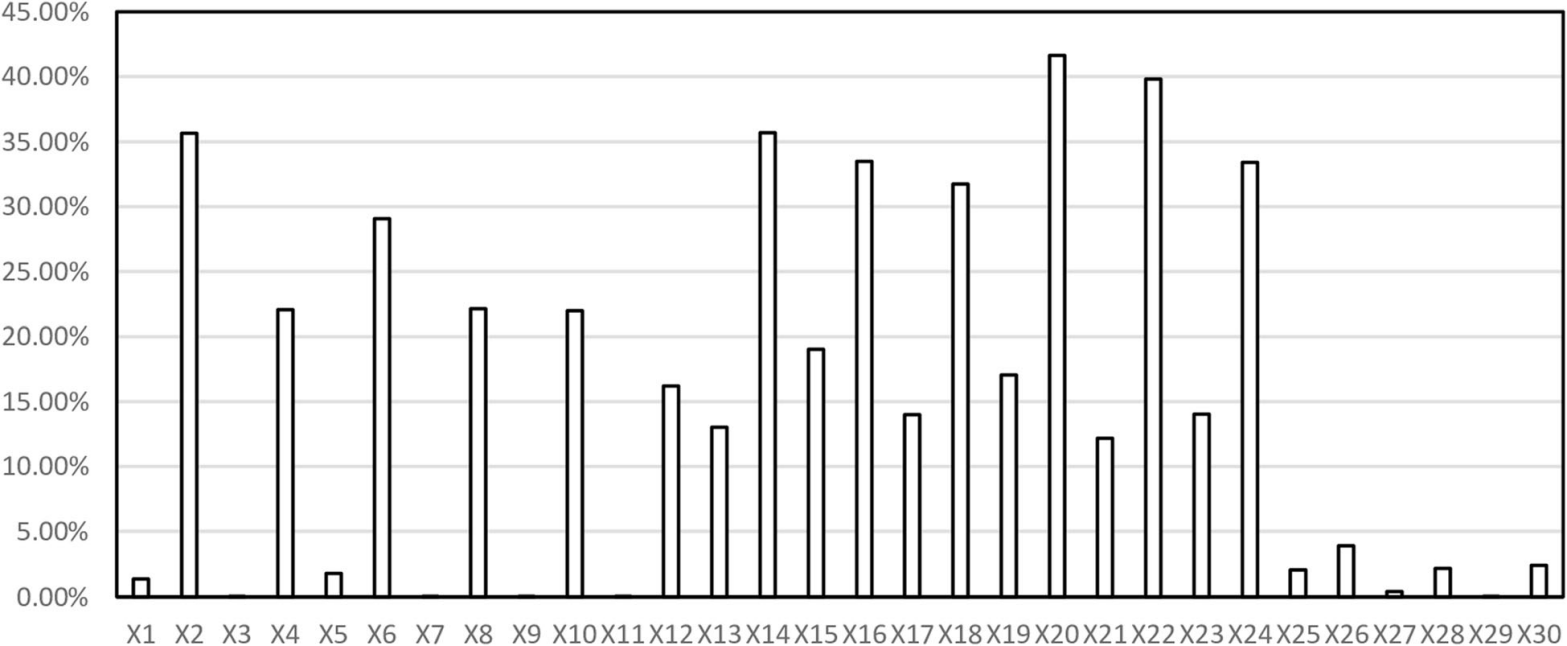
The technical and tactical diagnostic results of Harimoto

The results of the technical and tactical diagnosis analysis based on BP neural network model were shown in Fig. 5.

##### The perspective of game division (the beginning, the middle, and the end)

At the beginning of the game, the weight of × 20 was the largest, indicating that the SR of the fourth stroke had the greatest influence on the winning probability, followed by × 2, × 8, and × 14, which showed that the SR of the serve, receive, and the third stroke had a greater influence on the winning probability. The two technical and tactical indicators × 13 and × 19 had a certain impact on the winning probability. The indicators × 1, × 7, × 25, and × 26 had little effect on the winning probability.

In the middle of the game, the weight of × 22 was the largest, indicating that the SR of the fourth stroke had the greatest impact on the winning probability, followed by × 4, × 10, and × 16, which showed that these three indicators had a greater influence on the winning probability. The two technical and tactical indicators × 15 and × 21 had a certain impact on the winning probability. The indicators × 3, × 9, × 27, and × 28 had little effect on the probability of winning.

At the end of the game, the weight of × 24 was the largest, indicating that the SR of the fourth stroke had the largest influence on the winning probability, followed by × 6 and × 18, which showed that the SR of serve and the third stroke had a greater influence on the winning probability. The three technical and tactical indices × 12, × 17, and × 23 had a certain influence on the winning probability. The indicators × 5, × 11, × 29, and × 30 had little impact on the winning probability.

##### The perspective of the division of the three-phase evaluation method

At the serve and attack phase, the weight values of X2 and × 14 were large, indicating that the SR of the serve and the third stroke at the beginning of the game had the greatest influence on the winning probability, followed by × 4, × 6, × 16, and × 18, which showed that these four technical and tactical indicators had a greater impact on the probability of winning. The indicators × 13, × 15, and × 17 had a certain influence on the winning probability. The indicators of X1, × 3, and × 5 had little effect on the winning probability.

At the receive and attack phase, the weight of × 20 was the largest, indicating that the SR of the fourth stroke at the beginning of the game had the greatest influence on the winning probability, followed by × 22 and × 24, which showed that the SR of the fourth stroke in the middle and at the end of the game had a greater influence on the winning probability. The indicators × 8, × 10, × 12, × 19, × 21, and × 23 had a certain influence on the winning probability. The indicators X1, × 9, and × 11 had little effect on the winning probability.

At the stalemate phase, all the indicators × 25, × 26, × 27, × 28, × 29, and X30 had little effect on the winning probability.

## Discussion

The Double Three-Phase evaluation method of table tennis matches provides a more scientific and comprehensive perspective to analyze the competitive ability of athletes compared with the traditional three-phase evaluation method. The results of technical and tactical statistics, based on the Double Three-Phase method, are clearer and closer to the actual situation of table tennis competition, which better reflects the variation and strength of players’ techniques and tactics in the competition [12]. The results from this study indicated that using the Double Three-Phase evaluation method could produce a technical and tactical diagnosis model of table tennis matches employing a BP neural network and the model showed high prediction accuracy. Predicting with the generalized linear models is much simple, convenient, and easy to implement and understand, however, the fitting degree and prediction accuracy of the established model are not as good as that of the model based on BP neural network in table tennis, as that there is a nonlinear relationship between table tennis players’ sports quality and the competition results [7].

After establishing the diagnosis model, according to the requirements of the athlete’s technical diagnosis, a certain index value can be increased or reduced, then, input it into the neural network diagnosis model, the corresponding output value (winning probability) of the model can be obtained. Thus, it is easy to find the factors that have a significant impact on the competition result of a match and provides a theoretical reference for formulating a targeted training plan [29]. In this study, the diagnostic model was used to diagnose the techniques and tactics of Japanese famous table tennis player (Harimoto), and the results suggested that he should make full use of his advantage of active attack in the competition, and improve the quality of stalemate balls to make himself in a relatively dominant position and improve the winning probability, which supports the study of Chien et al. [31].

Although the technical and tactical diagnosis model of table tennis matches based on BP neural network had a high prediction accuracy and had a good ability to analyze the technical and tactical abilities of table tennis players, there still may be some difference between the output of the model and the true competition results. Several probable factors that may contribute to the difference were the: (a) training and test sample size. In this study, the training and test sample size was 70 and 30 respectively, which can be still increased for a higher model’s prediction accuracy [18], (b) selection of technical and tactical diagnosis indices. In the study, only 30 technical and tactical analysis indices were selected and the data were input into the model to calculate the output. There are also other factors influencing the player’s competition results such as psychological and environmental elements, the opponent, and so on [32], (c) the structure of the neural network model, including the number of hidden layers, the number of the hidden layer’s neurons, the training and transferring function, et al., which can all be changed according to the requirement of the model’s accuracy [33], and (d) the indicator winning probability can only partly reflect the competition result of a match. Hence, the output of the diagnostic model cannot fully represent the actual competition result.

It appears that the BP neural network can retain the mapping relationship between the index and the past competition result, and the technical and tactical level of athletes will change over time. When the technical and tactical level of the athlete changes, as long as the latest technical and tactical data are input into the BP neural network model, the new connection weights that reflect the current technical and tactical level of athletes can be obtained by retraining the existing neural network model. Therefore, it is necessary to track the athletes’ techniques and tactics for a long time to collect new technical and tactical data to retrain the model, thus the diagnostic model can be modified and maintain a good diagnostic ability.

## Conclusions

The technical and tactical diagnosis model of table tennis matches based on BP neural network had a high prediction accuracy and highly efficient in fitting. By using this model, the weights of the influence of athletes’ technical and tactical indices on the winning probability of the competition can be calculated, which provides a valuable reference for formulating targeted training plans of players.

For Harimoto, the technique of attack after receive has the greatest influence on the probability of winning, followed by the attack after serve. The stalemate technique has little effect on the winning probability.

### Implications

The diagnosis model can be used to analyze the technical and tactical characteristics of table tennis players and evaluate the strength of athletes’ techniques and tactics. After the weights of the influence of athletes’ technical and tactical indices on the winning probability of the competition are calculated, the indices that have a significant impact on the winning probability can be determined, which helps coaches to make targeted training and competition plan for table tennis players to improve the athletic ability of players.

For Harimoto, he should strengthen the training of the attack after serve and attack after receive skills, strengthen the consciousness of attack, and improve the quality of stalemate balls so as to increase the scoring rate and enhance the winning probability of competition.

### Limitations

In this study, only 100 matches of the world’s elite male table tennis players were selected. In the follow-up study, more matches can be selected as training samples to gain a better training and fitting accuracy of the BP neural network model. Besides, this study took only one player as an analyzing example, more players from different countries can be selected for a comparative analysis of their technical and tactical characteristics in the future study. Moreover, the technical and tactical indices did not involve specific technical action, so the suggestion proposed may not be targeted enough. Finally, the tests to confirm the linearity/non-linearity relationship between the competition results and players’ sports quality can be conducted in the future study.

## Supplementary Information

**Additional file 1: Appendix I.** The Matlab code for the evaluation of the diagnostic model. **Appendix II.** The Matlab code for the application of the model (“Harimoto” case).

## Data Availability

The datasets generated during the current study are not publicly available, but are available from the corresponding author upon reasonable request.
