# Supplementary material for: Technical and tactical diagnosis model of table tennis matches based on BP neural network
Source: BMC Sports Sci Med Rehabil. 2021 May 20;13:54. doi: 10.1186/s13102-021-00283-3 (PMC8136171; doi:10.1186/s13102-021-00283-3)
Supplement: Supplementary file 1 — Additional file 1: Appendix I. The Matlab code for the evaluation of the diagnostic model. Appendix II. The Matlab code for the application of the model (“Harimoto” case). [file 13102_2021_283_MOESM1_ESM.docx]

**Appendix I: The Matlab code for the evaluation of the diagnostic model**

all = xlsread('p_train.xls');

out=[];

for i=1:N

str=randperm(100);

str=str(1:70);

a=all(str,:);

a =a';

t_all = xlsread('p_target.xls');

t=t_all(str1,:);

t =t';

net = newff(minmax(a),[31,1],{'tansig','purelin'},'trainlm');

net.trainParam.show =5;

net.trainParam.epochs =300;

net.trainParam.goal =1e-5;

net = init(net);

[net,tr] = train(net, a, t);

b= sim(net,a);

c=abs(b-t);

c= sum(c)/length(c); % c represents mean error

[m,k,d]= postreg(b, t);

out=[out;c,d];

end

xlswrite('train_out.xls',out)

*Note.* The training parameter show is the times of each training before displaying the training results, the epoch is the number of training times, and the goal is the training error range between the output and actual value of the model.

**Appendix II: The Matlab code for the application of the model ("Harimoto" case)**

a = zeros(20,30)

a = xlsread('case_p.xls')

a =a'

t = zeros(1,30)

t = xlsread('case_t.xls')

t =t'

net = newff(minmax(a),[31,1],{'tansig','purelin'},'trainlm');

net.trainParam.show =5;

net.trainParam.epochs =300;

net.trainParam.goal =1e-5;

net = init(net)

[net,tr] = train(net, a, t);

b= sim(net, a)

b = b'

xlswrite('out.xls', b)

b =b'

[m,c,d] = postreg(b, t)

*Note.* The training parameter show is the times of each training before displaying the training results, the epoch is the number of training times, and the goal is the training error range between the output and actual value of the model.
